# Improving contraceptive agency through peer social support: findings from a longitudinal qualitative evaluation of the I-CAN intervention in Uganda

**DOI:** 10.3389/fgwh.2025.1544333

**Published:** 2025-05-23

**Authors:** Erica Sedlander, Beth Phillips, Isabelle Thapar, Catherine Birabwa, Lauren Suchman, Madeline Griffith, Dinah Amongin, Ronald Wasswa, Lynn Atuyambe, Jenny Liu, Peter Waiswa, Kelsey Holt

**Affiliations:** ^1^ Department of Social and Behavioral Sciences, School of Nursing, Institute for Health & Aging, University of California, San Francisco, San Francisco, CA, United States; ^2^ Department of Family and Community Medicine, University of California, San Francisco, San Francisco, CA, United States; ^3^ School of Public Health, College of Health Sciences, Makerere University, Kampala, Uganda

**Keywords:** contraception, agency, longitudinal evaluation, qualitative, sub-Saharan Africa (SSA)

## Abstract

**Background:**

Sexual and reproductive health organizations have been advocating for a human rights-based approach to contraceptive programming for many years, but progress has been slow. Peer social support shows promise to address structural and social barriers limiting women's agency to make and act on decisions related to contraception, but evidence-based models are lacking. Informed by Social Support Theory and the Contraceptive Agency Framework, we used human-centered design to develop “I-CAN”, a community-based peer mentorship intervention in which experienced contraception users in Uganda provide tailored support to peers to promote agency and self-efficacy to use self-injectable contraception among women interested in this method. We conducted a six-month pilot of I-CAN and report here on qualitative findings from a longitudinal study exploring I-CAN's social support mechanisms.

**Methods:**

We conducted serial in-depth interviews with *n* = 25 women who received mentorship at baseline, three months, and six months in 2023. We conducted parallel interviews with a comparison group (*n* = 15) without the intervention. Women were purposefully sampled for diversity in contraceptive use, district, and age. We analyzed interviews using a codebook informed by I-CAN's theory of action.

**Results:**

We identified two primary ways in which I-CAN peer social support appeared to improve mentee agency more than existing social support in the control group: (1) improved contraceptive knowledge, particularly allaying side effect concerns, and (2) improved ability to act on contraceptive preferences via communication with unsupportive partners, covert use, or accessing contraceptive services or products. Less prominent changes compared to the control included improved self-efficacy to self-inject and perceived control over and consciousness of the right to contraceptive choice.

**Conclusions:**

Underpinned by a human rights-based approach to contraception, the I-CAN intervention, shows promise that locally tailored peer social support models can effectively improve contraceptive agency, particularly related to knowledge and partner communication.

## Introduction

Myriad social and structural barriers, including prohibitive social norms (1, 2), lack of access to high quality contraceptive services (3, 4), and unaddressed concerns about contraceptive side effects (5–8), hinder women's agency to make and act on their contraceptive decisions. These barriers are compounded in rural areas of sub-Saharan Africa where there are additional healthcare access challenges and where inequitable gender norms are often intensified (9, 10).

Systematic reviews affirm the promise of peer support interventions across many areas of health (11, 12). Yet, there is a dearth of tested approaches to leverage experienced contraception users as a positive social support mechanism among women in rural sub-Saharan Africa (13). Qualitative evidence that social support from peers helps some young people overcome barriers to using contraception corroborates the potential of peer support models in this context (14, 15).

Further, the self-care technology subcutaneous depot medroxyprogesterone acetate (DMPA-SC) shows promise for helping rural women use contraception when and if they choose to (16, 17). Women can take home DMPA-SC units to store for self-administration after receiving training, and research across sub-Saharan Africa shows that women can not only feasibly manage this procedure (18–20) but that the option to self-inject may also help address barriers to use as continuation rates are higher compared to those who receive provider-administered DMPA-SC (21–23). However, uptake of self-injection of DMPA-SC has been low; prevalence in Uganda, for example, remains <1% despite offering this option since 2017 (24). Recent qualitative studies in Uganda document fear of injecting oneself and lack of consistent supply as barriers to women using DMPA-SC for self-injection (25, 26).

To address both the dearth of tested peer support models in the contraception field and documented barriers to use of self-injection of DMPA-SC, we developed the theoretically-informed I-CAN intervention with community advisory boards in two largely rural districts of Uganda using human-centered design (27, 28) The intervention was designed to: (1) increase women's contraceptive agency, defined as the ability to “make and act on decisions related to whether to do something to avoid or delay pregnancy and what, if anything, to do when they are not actively trying to become pregnant” (29); and (2) increase women's self-efficacy to use DMPA-SC self-injectable contraceptives such that women could confidently choose this method if they were interested (27, 28). I-CAN was implemented by community-based organizations that recruit, and train lay women to draw on their lived experience to “mentor” other women in their villages (“mentees”). The mentorship included various forms of social support including house visits, referrals to health centers, active listening, and discussions around how to make and act on their contraceptive decisions. Mentors complement the role of community health workers in rural areas of Uganda who are often overloaded with responsibilities and have limited time to provide tailored support for more complex challenges such as unsupportive partners; indeed Uganda's national plan for improving contraceptive access includes a focus on peer support models to complement community health workers' services (30, 31).

In April 2023–October 2023, we implemented a six-month pilot of the I-CAN intervention in predominantly rural districts in Uganda. We used mixed methods to evaluate it (quantitative results will be published separately). This paper reports the qualitative results of our research question: How and to what extent does the informational, instrumental, and emotional support provided by I-CAN mentors change contraceptive agency and support people to use the new self-injectable contraceptive method when they are interested? We used serial in-depth interviews at three time points - before (baseline), mid-way at three months (midline), and after the intervention at six months (end line) - to examine women's experiences with the six-month intervention and compared them to a cohort from neighboring communities receiving their usual, non-intervention social support.

## Theory of action

**Figure 1:**
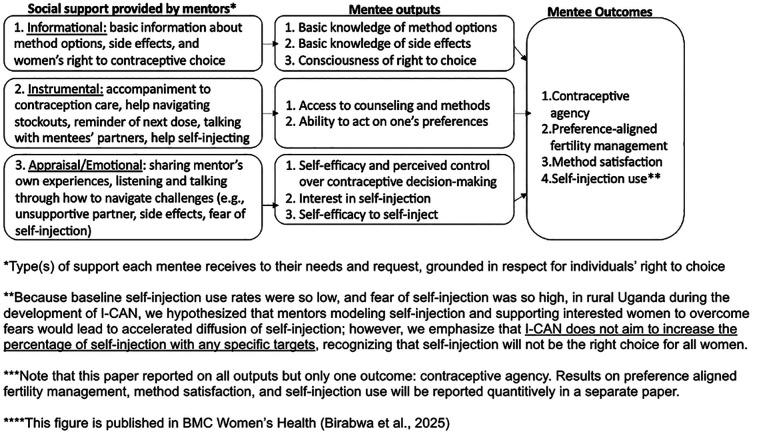
I-CAN theory of action.

I-CAN's theory of action draws on Social Support Theory and the Contraceptive Agency Framework (29, 32). Figure 1 I-CAN Theory of Action depicts the theoretically informed pathways by which we designed I-CAN to influence participants' (“mentees”) contraceptive agency and self-efficacy to self-inject DMPA-SC via different types of social support (28). The contraceptive agency framework was published to guide development of contraceptive programming and program evaluations that center a rights- and empowerment-based approach (29). Contraceptive agency is defined as one's ability to make and act on their own contraceptive preferences and decisions. Domains within this framework include (1) being clear about one's personal values, (2) having information and support in accordance with one's preferences, (3) being conscious of the right to contraceptive choice, (4) exercising critical reflection, (5) believing one has control over contraceptive decisions, (6) having self-efficacy to form and act on contraceptive preferences, (7) being able to act in accordance with one's preferences related to contraception, and (8) having control over who and to what extent others are involved in decisions (29).

We developed I-CAN to address specific barriers to contraceptive agency identified in our community-engaged, human-centered design process and determined areas of support to focus on; these included informational, instrumental, and emotional support (28). Past research has shown that social support can serve as a key “protective” factor that reduced individuals' vulnerability to the deleterious effects of stress on health (33). The I-CAN intervention introduces a new social network member to women's lives, a contraception mentor, with experience using myriad methods including self-injection (34). This mentor was in addition to any existing support system that the women had (e.g., community health workers, friends, family members, etc.). Mentors identified women interested in receiving contraceptive peer support in their community through outreach and provided social support tailored to what each woman wanted, including consultations at women's homes, clinic accompaniment, and referrals to see healthcare providers. I-CAN informational support includes providing basic information related to various methods and potential side effects, instructional materials for self-injectable contraception, and information about women's right to contraceptive choice (28). Instrumental support includes accompaniment to contraception care, help navigating stockouts, reminders, talking with women's partners, and help self-injecting. Emotional support includes a mentor listening and talking through how to navigate challenges and sharing their own contraceptive experiences. See (28) for a full description of the intervention design process and the I-CAN theory of action. See Figure 1. I-CAN Theory of Action below which depicts each form of social support and how it relates to contraceptive agency.

It is important to note that this intervention included support for women to choose among a range of contraceptive options; given low rates of self-injection uptake despite the potential of this novel self-care technology, this method option was a particular focus. “Mentees” opted-in to get tailored support (in the form of one-on-one consultations and/or accompaniment to health services). Mentors were trained to provide neutral support without bias for any specific contraceptive method, including the decision not to use contraception, and respect women's decisions without being directive.

## Materials and methods

### I-CAN pilot

The community-based organizations that participated in the co-design process of the I-CAN intervention, Baitambogwe Community Health Care Initiative (BACHI) and AIDS Information Center (AIC), piloted the I-CAN intervention in Mayuge and Oyam districts for six months from April to September 2023. BACHI and AIC recruited and trained women to be peer mentors. Eligibility criteria for being a mentor included: having prior experience with at least two contraceptive methods, including self-injection; having self-injected themselves at least two times; and planning to live in their village for the duration of the implementation period. Mentors were recruited through advertisements posted in public places (e.g., health centers, markets, village council offices), announcements on radio and in women's groups, and through referrals from village council members and community health workers. Mentors underwent a four-day training that included modules on person-centered and rights-based family planning principles and Social Support Theory (32), followed by interactive modules where mentors practiced conducting outreach. Mentors volunteered their time and received a stipend of ∼$10USD/week, branded t-shirts and backpacks, and supportive supervision every month.

### Pilot evaluation

In this manuscript, we report on the longitudinal qualitative evaluation of I-CAN's effectiveness, consisting of serial in-depth interviews with 25 mentees and 15 women in comparison sub counties in the Eastern and Central North regions of Uganda. Interview data were collected at three timepoints (baseline, three months, and six months).

### Ethical approval

This study was approved by Makerere School of Public Health (MakSPH) Research and Ethics Committee (SPH-812), Uganda National Council for Science and Technology (HS1087ES) and University of California, San Francisco (UCSF) Institutional Review Board (UCSF #21-33997).

### Study sites, sampling, and recruitment

Study sites included six sub-counties within two largely rural districts in Uganda: The Mayuge district in Eastern Central Uganda, and Oyam district in Northern Uganda. Intervention sub-counties were selected by the implementing partners and design community advisory boards to include one urban subcounty and one rural subcounty in each study district. The comparison subcounty was selected for being geographically distinct (i.e., not neighboring either study subcounty) with comparable population demographics to the intervention sites.

Eligibility criteria for participants in this mixed methods pilot included being a woman of reproductive age (15–45 years). Mentees and women from the comparison group were purposefully sampled to ensure roughly equal representation by contraceptive use (non-users, contraceptive users broadly, and self-injection users), district, and age (15–19 vs. 20–45 years).

Twenty-five mentee evaluation participants were chosen from the total of 302 mentees delivering the pilot intervention based on study inclusion criteria, listed above. Mentees were recruited for interviews with help from the mentors and women in the comparison group were identified with help from the Village Health Team members, volunteers who provide health information and link community members with available health services. To determine contraceptive use categorization, at the time of recruitment mentors asked women what they were doing to prevent pregnancy and noted this in their enrollment logs. Research assistants then received this information from community-based organizations to help with study recruitment.

### Instrument

The research team developed the interview guides by drawing on the Contraceptive Agency Framework (29) to probe about different components of agency related to contraceptive decisions and actions. The guides included questions on how women make decisions about contraception and their experiences accessing and using contraception. Guides for the intervention group additionally probed on experiences over the course of the pilot intervention to evaluate women's experiences longitudinally over six months.

### Data collection procedures

Prior to each interview, the research team obtained written informed consent from selected participants. Interviews were conducted in Langi (Oyam) and Lusoga (Mayuge), the dominant local dialect in the pilot areas. Mentors were experienced contraceptive users from the community who were trained in the I-CAN mentorship program to provide support to peers. Interviews were conducted in private spaces, such as a woman's house or near a school or health facility, and each interview was recorded and lasted 60–90 min. The research team made intentional efforts to safeguard mentees' privacy and confidentiality. For follow up interviews at three months (midline) and six months after the intervention (end line), research assistants received support from the mentor to schedule an interview.

### Analysis

Trained transcriptionists who were native speakers of the local languages and fluent in English transcribed and translated each interview. Four researchers (ES, BP, IT, MG) coded the transcripts in Dedoose using the codebooks informed by the Contraceptive Agency framework and Social Support Theory (29, 32). After coding each interview, we wrote memos pulling out emerging themes, extracted code reports, and created a list of priority codes, and wrote code summaries based on the code reports. Next, we reviewed the completed memos across the three interview time points (baseline, midline, and end line) and mapped emerging themes onto the contraceptive agency framework with two domains and six subdomains. As we reviewed memos, we noted any change in any of the outputs from the theory of action (binary yes/no). Subsequently, we created a matrix in Excel and extracted relevant quotes for each of the theory of action constructs for both the mentees and comparison group interviewees.

Primarily, we sought to understand whether the hypothesized pathways in the theory of action (Figure 1) appeared to be working as intended and hypothesized. Secondly, we examined which components of the pilot intervention were most beneficial to increasing contraceptive agency and mentees' self-efficacy to self-inject. After we coded each interview transcript, we generated a memo to highlight key themes related to the research questions' domains. Once we completed all coding and memos, we analyzed memos and summarized all relevant code reports, allowing for pattern recognition in the data. We subsequently mapped key themes from the memo analysis and code summaries onto the contraceptive agency framework.

## Results

The study included a total of 40 participants, *n* = 25 women in the mentee group and *n* = 15 women in the comparison group. Demographic characteristics of respondents are summarized in Table 1: Description of the Sample. The comparison group is missing some information (*n* = 8) on the employment status among the comparison group in Mayuge. Although we planned to conduct a total of 120 interviews spaced three months apart, two women in the comparison group were lost to follow-up at three months, three comparison respondents were lost at six months, and one mentee was lost six months, resulting in 114 interviews total.

**Table 1:** Description of the sample (*N* = 40).

		Mentees						Comparison					
		Mayuge		Oyam		Overall		Mayuge		Oyam		Overall	
		*n* = 12		*n* = 13		*N* = 25		*n* = 8		*n* = 7		*N* = 15	
Age	15–19	5	42%	3	23%	8	32%	4	50%	2	29%	6	40%
	20–45	7	58%	10	77%	17	68%	4	50%	5	71%	9	60%
Contraceptive use status at baseline	Current non-user	6	50%	9	69%	15	60%	6	75%	3	43%	9	60%
	Current user	6	50%	4	31%	10	40%	2	25%	4	57%	6	40%
SI use status at baseline	Never self-injected	11	92%	12	92%	23	92%	4	50%	5	71%	9	60%
	Has self-injected	1	8%	1	8%	2	8%	4	50%	2	29%	6	40%
Marital status	Married	7	58%	9	69%	16	64%	5	63%	7	100%	12	80%
	Partnered/cohabitating	5	42%	4	31%	9	36%	3	38%	0	0%	3	20%
Education	Less than secondary school	10	83%	9	69%	19	76%	6	75%	5	71%	11	73%
	Secondary or higher	2	17%	4	31%	6	24%	2	25%	2	29%	4	27%
Employment	Employed^a^	0	0%	6	46%	6	24%	–^b^	–^b^	6	86%	–^b^	–^b^
	Unemployed	12	100%	7	54%	19	76%	–^b^	–^b^	1	14%	–^b^	–^b^
Religion	Protestant	5	42%	6	46%	11	44%	0	0%	0	0%	0	0%-
	Catholic	6	50%	5	38%	11	44%	2	25%	3	43%	5	33%
	Pentecostal	1	8%	1	8%	2	8%	0	0%	0	0%	0	0%
	Other	0	0%	1	8%	1	4%	6	75%	4	57%	10	66%

^a^ “Employed” defined as having done work for pay or work that provides compensation other than cash in the past month.

^b^ Data incomplete (–) due to logistical constraints.

Social support from mentors appeared to improve contraceptive agency in two primary domains: (1) informational support, which improved respondents' contraceptive knowledge by empathetically discussing concerns about side effects including fears of infertility and bleeding changes and (2) instrumental support by talking directly with mentees' partners or helping them navigate covert use, which enabled mentees to act on their contraceptive preferences. Less prominently felt changes in contraceptive agency in the intervention group included increased self-efficacy to self-inject, a sense of control over contraceptive decision-making, and increased consciousness of the right to contraception choice.

### Mentors were perceived to improve contraceptive knowledge by empathetically discussing concerns about side effects, including fears of infertility and bleeding changes

Overall, mentees in the I-CAN pilot trusted the information that their mentors provided over information that they heard in the community. For example, one mentee said that she trusted the “good” information from her mentor above the community because, “sometimes the other people don’t even know what they are talking about, they just say anything they are not even sure about” (mentee, Oyam, age 24). Another mentee said that her mentor helped her to “demystify all the lies people used to tell us about family planning”. On the contrary, women from the comparison group expressed that they wished they had someone they could trust to discuss myths in the community “because the things people say in the community and the things people say at the health facility is very different” (mentee, Oyam, age 21).

While many women in the comparison group wanted more information about side effects, some of the most valuable informational support from mentors addressed the ubiquitous fear that contraception causes infertility, “makes the uterus rot”, and “spoils women”. It was evident in respondents' transcripts that the information that contraceptives do *not* cause infertility made women feel “safe” and “motivates them” to use it. One mentee respondent mentioned how she was able to make an informed decision about family planning after getting this information from her mentor, “I had no trust in family planning, even my mother-in-law told me that she stopped producing after receiving a family planning injection, so I believed that you could not conceive after getting a family planning injection. But when I went to this mentor, she gave me the right information, then I made my decision” (mentee, Mayuge, age 23).

Mentees reported that mentors also provided support around other side effects like bleeding such as saying, “if it [contraception] treats you badly, you can change it” (mentee, Mayuge, age 29) and allayed fears about associated changes in menstruation. Mentors assured mentees that these changes are a normal part of using contraception, like the mentee who recalled “I used to pour a lot of blood [… but the mentor] she told me that you be strong you will see when you are better” (mentee, Mayuge, age 21).

While a few women in the comparison group received “support”, “advice”, and “encouragement” to start using contraception from friends, family, and information from the health center we did not find any instances of women in the comparison group receiving *informational support* from their existing support networks addressing concerns about bleeding-related side effects or that contraception causes infertility. One woman in the comparison group stated that she wanted more information about contraceptive methods and creators of the method to improve them because the “side effects are too much” (woman from the comparison group, Oyam, age 16).

In contrast, women in the *comparison group* described receiving informational support primarily from health center staff who spoke to them about contraceptive options and managing side effects, but in a way that was more one-sided without tailored support. One woman from the comparison group said, “The health workers came and taught and told us the good things and ease in using it. They gathered us in the center and taught us. And even the Village Health Teams from the community continued teaching us and I saw that they told us the same information, I went for it” (woman from the comparison group, Mayuge, age 25). At end line, the same woman discussed how she sought advice from the health center when she was starting a new method and was inquiring about side effects, “I have never bled, so I don’t know whether this can cause me problems”. In response, the health workers advised her saying, “bleeding is not a problem” (woman from the comparison group, Mayuge, age 25).

While some women in the comparison group felt like they had enough *informational support* from health center staff, others wished that they had someone they could talk to about side effect management. One woman stated, “The side effects are too much - especially the bleeding. You will find that, if you are bleeding non-stop, the man even becomes impatient with you, and he will start having other relationships outside which defeats the purpose really” (woman from the comparison group, Oyam, age 23). Similarly, three women in the comparison group said that when they got new methods at the health center (implant and IUD), they had questions about side effects but the providers at the public health facility were so “busy”, they just got the method without asking and left.

### Instrumental support from mentors enabled women to act on their contraceptive preferences

#### Mentees reported instrumental support related to navigating contraceptive conversations and use with partners

Among women in the intervention who wanted to use contraception but had non-supportive partners, many mentees shared that mentors provided women with *instrumental support* by helping them navigate conversations with partners and convince their partners that contraception was a smart choice. Mentees also stated that mentors did this by either “guiding” women on what to say to their partners or “talking” with their mentees' partners directly “which has been very helpful in making my decision [to use contraception]” (mentee, Oyam, age 23). Several mentees noted that they were able to change their partners' minds about contraception over the course of the intervention and subsequently made the decision to start using it*.* The shift in decision making power between partners in several mentees' relationships demonstrates how increasing women's contraceptive agency can begin to address some gender-related barriers to contraceptive use.

Mentees noted that their other support systems, including sisters, aunties, mothers and healthcare workers, whom some relied on for informational support about contraceptive methods, could not be trusted to give them guidance on how to manage conversations about contraception within their relationships. As one mentee explained in her six-month interview, “When I talk to her [mentor], I feel comfortable compared to when I talk to others. My mentor may keep [our conversation] secret, but I don’t trust others to keep it secret” (mentee, Mayuge, age 20). For example, one woman said, “I would ask my husband, but he would refuse [to let me use family planning], but after some time, he understood. The mentor also talks to him, and it helped him to understand” (mentee, Oyam, age 23). And another woman said, “it has changed so much [how she communicates with her husband about family planning], because the mentor talks to us in a careful gentle way to help us to understand, so I have also learnt that, to be able to talk to my husband, I have to be gentle and careful with the way I approach him with issues” (mentee, Oyam, age 38).

Among women whose partners were not supportive, mentee respondents indicated that mentors helped them *act on their contraceptive preferences* by helping them use contraception covertly. The fact that mentors came from the community helped maintain confidentiality; talking to other women from the community was less suspect than traveling to the health center. Oftentimes, mentors would provide *instrumental support* by bringing contraception directly to the women at no cost. For some mentee women who were using covertly, that was the difference between being able to use contraception or not. For example, one woman at baseline mentioned that she gets all her money from her husband. So if she spends any on contraception, or uses certain methods like the IUD, he would know, which was one reason why she sought a more covert method like injections. Over the course of the intervention, she moved from using contraceptives covertly at baseline to telling her husband and “seeking permission” after three months and her husband “feeling good” about how the mentor program has helped her. By the six-month follow-up interview, she said that she spoke with her husband because “the challenges that happen to me he has to know, he is the one to help me when the mentor is not around” (mentee, Mayuge, age 24). Another mentee mentioned that she felt that deciding for herself gave her “control when to conceive and not”. At six months, she wanted to “get protected” because she was still in school and wanted to “protect” against getting pregnant in the future (mentee, Mayuge, age 19).

Women in the comparison group noted sisters, aunts, mothers, and sometimes female friends who they rely on for instrumental support, especially when navigating contraceptive use with unsupportive partners. One woman in the comparison group discussed how she goes to her “auntie” to discuss whether she should start family planning, and how she encouraged her to speak with her unsupportive husband. She said, “[my auntie] advised me to go and talk to my husband, because I could easily get pregnant” (woman from the comparison group, Oyam, age 16). However, women in the comparison group did not describe any assistance around how to talk to unsupportive partners or offers to speak directly to them.

#### Mentees reported that mentors provided instrumental support to help women access the healthcare system

Many mentees expressed that mentors provided *instrumental support* to mentees by reminding them when and where to get their next injection and providing them with referrals to the health center. Mentees using self-injectables found it valuable that their mentor reminded them about when to take their next dose within the context of their busy lives as described by one woman, “these days there is a lot of work here in the village, so, it is easy to forget, but she keeps reminding us” (mentee, Oyam, age 30). Some mentees appreciated the mentor accompanying them to the health center, as recounted by this mentee in her three-month interview, “if I had gone alone, I wouldn’t accomplish what had taken me there since I didn’t know where to start from and what to do” (mentee, Oyam, age 20).

Other mentors referred mentees with various needs to the health center (oftentimes with a formal written referral from the I-CAN mentor) as this mentee describes, “The next injection she [the mentor] told me to go and pick the [DMPA-SC self-injectable] kit from up there [village clinic]. So, I went there, I gave the lady a referral note; she [the mentor] gave me, the lady gave me the kit and when she gave me, I immediately injected myself there and then” (mentee, Mayuge, age 22). In a few instances, in part thanks to the mentor referrals, women switched methods and their side effects subsequently improved.

In contrast, women in the comparison group did not have existing people outside of the providers in the healthcare facility to provide them with instrumental support in their contraceptive decisions, including navigating the health center and appointments or how to manage side effects.

#### Stock out issues hindered contraceptive agency in both intervention and comparison groups

In both comparison and mentee groups (and in both Mayuge and Oyam), there were issues around contraceptive stockouts and for self-injection, sometimes women were only able to get one dose hindering their ability to use contraception and removing the convenience and privacy of self-injecting at home. In some instances, mentors were able to address stock outs by suggesting mentees visit a different health center, but in others, women did not have the method of their choice (or enough of it). One woman in the comparison group said, “The services concerning family planning that I need are the drugs. If I can have them, I don’t have to walk to the hospital but can be at my home and I administer it to myself” (woman from the comparison group, Mayuge, age 25). Another woman in the comparison group said, “if I don’t have the accurate knowledge on when and where to access these services, even for follow ups and for refills, then I fear to start using it” (woman from the comparison group, Oyam, age 21). Most respondents noted that a main way mentors could continue to help them self-inject was by ensuring a readily available supply of DMPA-SC.

### Instrumental and emotional support from mentors increased mentees' self-efficacy to self-inject, adding an extra layer of support for mentees

This theme resulted in more minor changes in contraceptive agency compared to the first two themes. Nonetheless, mentees reported that mentors demonstrated how to self-inject (with a training kit with a needle/syringe) to some women (both *instrumental support* with specific steps on how to insert the syringe and *emotional support* to overcome fear of self-injecting among those interested in self-injection) and this skill increased their self-efficacy or confidence to self-inject. In other words, mentors' caring discussions with mentees and encouraging support helped women make the decision to use self-injection by making sure they were confident enough to choose the method if they desired to and supporting women overcoming psychological barriers, including fear of self-injectable contraception. One mentee woman said, “naturally, I am a very fearful person, but she taught me how to self-inject and I am doing it very well, on my own”. (mentee, Oyam, age 38). And another Oyam mentee (age 23) said, “I was encouraged, and trained on how to self-inject and now, I can inject myself any time I am due”.

Some mentees even felt like they could advise other women to self-inject, including an Oyam mentee who stated, “I can tell [a friend/relative about family planning]… because from what the mentor told us, I know” (mentee, Oyam, 38).Another Mayuge mentee said, “I also feared the injection but now I can inject myself, even my neighbor used to fear but after seeing me injecting myself she also tried, so, I taught her, and she also gained confidence and she is now also self-injecting”.

In the comparison group, some women felt that being “trained” to self-inject from “someone in the community” would help them overcome their fear and enable them to potentially inject themselves in the future. When asked by an interviewer what would make her sure of the injection method, one woman stated, “I would like to be taught more so that I can inject myself like a health worker without shaking” (woman from the comparison group, Mayuge, age 21).

Like the mentee group, a few women in the comparison group also reported that the village health center taught them to self-inject. These women, like those in the mentee group, expressed eagerness to teach others and confidence in their ability to continue to inject in the future and to support other women to also inject themselves. For example, one woman said at six months, “At first I used to quake but whenever I got used to it, this time I am confident, and I can inject myself without quaking” and she “doesn’t have any challenges getting family planning services” at the health center (woman from the comparison group, Mayuge, age 21).

Discussed by many women in the comparison group, it was clear that even in the absence of the mentorship program, once women tried and successfully self-injected, they felt a sense of confidence. One woman in the comparison group said, “I have discussed with many people because when I started using it [self-injection], I came out boldly as a user, so I received the training, and now as I talk, I can even inject someone with a contraceptive” (woman from the comparison group, Oyam, age 21). But unlike in the mentee group, self-injection support was rare and brief.

One woman said, “I received some support [around self-injection] because I came and they [healthcare provider] gave me training”.

Interviewer: “anything else, or any other support?”

Respondent: “just the teaching that was given to people. They were talking about the benefits of family planning” (woman from the comparison group, Oyam, age 21).

Additionally, women in the comparison group did not receive the degree of instrumental and emotional support mentors provided to mentees to navigate use with unsupportive partners. For example, one woman in the comparison group had to stop using contraception altogether when her husband found out she was covertly self-injecting. At her six-month interview, she had not gotten her period in four months, suggesting she was pregnant. She said, “It is my husband who made the decision…I still wanted to use [self-injection] but my husband made a decision. He doesn’t want family planning” (woman from the comparison group, Mayuge, age 23).

### Mentees reported that mentors gave women a slightly improved sense of control over contraceptive decision-making

This theme, in addition to theme three, resulted in more minor changes in contraceptive agency compared with the first two themes. Some mentees described that mentors supported them to have more control related to contraceptive decision-making. For example, one woman mentioned that the program enabled her to “make my own decisions now, and I also have someone that I can go and talk to if I have a problem” (mentee, Oyam, age 24). We noted that most women were aware that their husband was the “head of the household” (mentee, Oyam, age 21) and had control over whether they use contraception, “He is the one that controls me” (mentee, Mayuge, age 29). In one instance, a 17-year-old was a previous contraceptive user and a non-user at baseline as she was told not to use contraception and had frequently heard in the community that contraception can “cause you not to give birth” and “your womb may fall out”. However, over the course of the intervention, she talked about contraceptives with her mentor, who offered advice from their own experiences, information about various contraceptive methods, and dispelled misinformation to her mentee and also with her mentee's boyfriend. The mentee discussed how she now felt confident in making her own decisions at end line saying the program has helped her feel supported in her choice “not to conceive”. At end line she and her boyfriend used condoms that he purchased for them from the health center (mentee, Mayuge, age 17). Similar to some other mentees, she experienced a shift in power dynamics within her relationship, enabling her to make and act on her contraceptive preferences and decisions. Some mentees felt more empowered to communicate their preferences with their male partners. For example, one mentee said, “I have now become solid, I had some fears back then, but now, I make my own decisions. I just talk to [my husband], and I proceed with what I want to do” (mentee, Oyam, age 20).

We found one instance in the comparison group, where a woman changed from getting advice and permission from her husband and village health center staff at baseline, to engaging in a conversation with her husband to make joint decisions and accepting her contraceptive use at midline, to deciding on her own to use her preferred family planning method at her six-month interview. “Previously, I used to [always talk to my husband and the health facility staff], but now, I have made up my mind…I don't ask anymore…because I now know what to do” (woman from the comparison group, Oyam, age 21).

In contrast to the intervention group and the role of the mentors, women in the comparison group did *not* mention that their peers bolstered their confidence to make decisions about contraceptive uptake. As previously mentioned, some women said that they received informational support from friends or family, but they did not say that it increased their sense of control over contraceptive decision-making.

### Mentors minimally contributed to igniting women's consciousness of the right to contraceptive choice

Mentors were trained to provide informational support around *consciousness of the right to contraceptive choice*, but evidence of this change emerged infrequently. However, we noted a few instances, including this one: “I don’t have any fears [of being seen accessing family planning services], there is no one I fear…because it is my right. [The mentor] encouraged me to just go [to the health center] as long as I need the help” (mentee, Oyam, age 36).

At baseline, one mentee stated that her husband “didn’t want her to use [contraception], because it makes women “barren””. She said that “[my husband] was telling me that after we have spaced, we should also have another child, but I haven’t agreed because it is my right to use that contraceptive”. At end line, she said that the mentor impacted her husband's perspective, and he was encouraging her to use it. She shared, “[the program] changed my life, given me confidence to talk to my friends [about contraception], which is something I couldn’t do before”. She also expressed how she is confident and “made up [her] mind” about her chosen method because it is “working well” for her (mentee, Oyam, age 21).

In contrast, we did *not* find any instances in the comparison group of change in consciousness of the right to contraceptive choice despite asking about decision making and contraceptive use at all three time points.

## Discussion

Using human-centered design, our team developed the I-CAN intervention with the goal of improving contraceptive agency and increasing self-efficacy to self-inject among women interested in this contraceptive method (28). Our qualitative findings from a six-month pilot of I-CAN identified several ways in which social support from mentors appeared to improve contraceptive agency among I-CAN participants compared to usual support among women in the comparison group. The primary changes, we observed qualitatively, were improved contraceptive knowledge and improved ability to act on contraceptive preferences.

Our findings align with a recent longitudinal qualitative study (not based on an intervention) that captured the changing nature of family planning needs, attitudes and behaviors, and similar to our study, found that discussions with peers is necessary to help women and couples meet their family planning needs (35). Our findings suggest that the I-CAN intervention amplified the natural potential of peer support by inviting peers to discuss family planning with other women from their community (peers/mentees) to support them in their family planning journey.

We posit that a key reason I-CAN was feasible, acceptable, and qualitatively showed improvements in contraceptive agency was that it was culturally appropriate and tailored to the context given our use of human-centered design to develop the intervention. Intervention materials—including training and implementation tools used by the Village Health Team, midwives, and nurses—were developed to be relevant for our specific study sites and with extensive input from our community advisory board. The solutions were grounded in the needs identified among community members in the pilot communities—for example, the human-centered design process revealed that, for these largely rural settings in Uganda, local members of the community would be the most appropriate to deliver the intervention for several reasons. Firstly, mentors could more easily make house visits to share contraceptive *information*, and the information was trusted as mentors were respected members of the community. Additionally, the training they received made women feel information coming from the mentors was especially trustworthy. Secondly, mentors were equipped, during their initial training and ongoing supportive supervision, to use culturally relevant strategies (*instrumental support*) to get women's partners onboard with contraceptive use when partners were not supportive, including offering their personal experience about their journey with contraceptives. Thirdly, as members of the community who use the same health centers, mentors were able to provide referrals to local health centers, and help women navigate stock outs by knowing which health center had the contraceptive method they wanted. Finally, they provided *emotional and instrumental support* to overcome fear of self-injection (among those interested in self-injection) by sharing their personal story which seemed attainable as they were from the same community.

Structural challenges appeared to limit I-CAN's ability to improve women's contraceptive agency in certain areas. For example, contraceptive stockouts posed challenges to women acting on their preferences during the pilot period as other research has shown (3, 4). Secondly, gender norms make it difficult for women to make their own decisions about contraceptive use (2, 36). Other work generated by our research team has similarly found that unequal gender power dynamics have a strong influence on women's ability to make contraceptive decisions in line with their own preferences (10). These inequitable gender norms may have impacted women's perceived control over contraceptive decision-making (one contraceptive agency subdomain). Indeed, in several cases, mentors spoke directly to the mentee's husband to get “permission”, or mentors helped mentees prepare to have their own discussions with their partners. This support enabled women to act on their contraceptive decisions, but we found less change in mentees' perception that they have control over their contraceptive decision-making compared to changes in other contraceptive agency subdomains. This type of change likely requires including men in the intervention to address inequitable gender attitudes and beliefs. Women may want shared decision making with their partner as found in other research from our team in Uganda (26). Additionally, some respondents (especially in the comparison group) mentioned that they needed to hide contraception from their in-laws.

## Limitations and strengths

Our study has some limitations that may affect the interpretation of our results. First, we are missing some information on the comparison groups' employment in Mayuge (see Table 1) which limits our ability to characterize the full sample, but it's unlikely to undermine our results. Additionally, the number of women who self-injected at baseline was higher in the comparison than in the mentee group, because some women who reported never self-injecting in the comparison group during baseline recruitment revealed in their baseline interview that they had at some point tried it. This could skew self-efficacy comparisons. For example, because self-injection in the mentee group was lower at baseline, we could interpret change to be higher because there is more potential for improvement. Furthermore, our focus on largely rural areas in Uganda means that our findings may need to be tailored for urban or peri-urban environments.

Despite these limitations, our study has several strengths including our longitudinal, qualitative cohort design which included three time points enabling us to show how contraceptive agency changed within one unit of analysis (the mentee) and the comparison group helped highlight the counterfactual – what would have happened in the absence of the mentor program. Additionally, the fact that the I-CAN program was designed to promote contraceptive agency—a rights-based concept—rather than contraceptive use makes the results from this pilot evaluation particularly valuable. Sexual and reproductive health organizations have been advocating for a human rights-based approach to contraceptive programming for many years (37–39) yet programming and evaluation approaches that center the long-term goals of women's empowerment and gender equity rather than shorter-term outcomes like taking up contraceptives (39) have lagged behind.

## Conclusions and policy and program implications

The I-CAN intervention shows promise that locally tailored peer social support models can effectively complement community health workers' role and improve contraceptive agency and increase self-efficacy to self-inject among women who are interested. Additionally, peer support with community members or community health workers is likely to be less expensive than interventions that involve medical providers, which further points to the potential sustainability of our human-centered, peer-focused design. Future research could adapt the intervention for other regions within Uganda or countries within sub-Saharan Africa and conduct further assessments of the large-scale effectiveness and sustainability of the program. Additionally, we found more change in some sub domains compared to others which could be explored further in subsequent interventions. Follow up studies could also explore if an extended or heavier dose intervention could improve control over contraceptive decision-making and consciousness of right to contraception choice in a more salient way. It is important to recognize that individual-level peer support models can only go so far to supporting women's contraceptive agency and that work to address inequitable gender norms by including men in the intervention (e.g., male partner or couples workshops) or other structural challenges to contraceptive access are also critical (10, 40).

## Data Availability

The datasets presented in this article are not readily available because this is a qualitative study using transcripts as the dataset. We are not making the transcripts publicly available but are open to partnering on subsequent papers. Requests to access the datasets should be directed to erica.sedlander@ucsf.edu.
